# Exogenous Ketones Lower Blood Glucose Level in Rested and Exercised Rodent Models

**DOI:** 10.3390/nu11102330

**Published:** 2019-10-01

**Authors:** Csilla Ari, Cem Murdun, Andrew P. Koutnik, Craig R. Goldhagen, Christopher Rogers, Collin Park, Sahil Bharwani, David M. Diamond, Mark S. Kindy, Dominic P. D’Agostino, Zsolt Kovács

**Affiliations:** 1 Department of Psychology, University of South Florida, Tampa, FL 33620, USA; crpark@mail.usf.edu (C.P.); sahilbharwani2692@gmail.com (S.B.); ddiamond@usf.edu (D.M.D.); 2 Department of Molecular Pharmacology and Physiology, Morsani College of Medicine, University of South Florida, Tampa, FL 33612, USA; biocem@gmail.com (C.M.); akoutnik@health.usf.edu (A.P.K.); goldhagencraig@gmail.com (C.R.G.); crogers@health.usf.edu (C.R.); ddagosti@health.usf.edu (D.P.D.); 3 Department of Pharmaceutical Sciences, College of Pharmacy, University of South Florida, Tampa, FL 33620, USA; kindym@health.usf.edu; 4 James A. Haley VA Medical Center, Tampa, FL 33612, USA; 5 Shriners Hospital for Children, Tampa, FL 33612, USA; 6 Institute for Human and Machine Cognition, Ocala, FL 33471, USA; 7 Savaria Department of Biology, ELTE Eötvös Loránd University, Savaria University Centre, Károlyi Gáspár tér 4., 9700 Szombathely, Hungary

**Keywords:** exercise, blood glucose, blood ketone, exogenous ketone supplements, ketogenic diet

## Abstract

Diseases involving inflammation and oxidative stress can be exacerbated by high blood glucose levels. Due to tight metabolic regulation, safely reducing blood glucose can prove difficult. The ketogenic diet (KD) reduces absolute glucose and insulin, while increasing fatty acid oxidation, ketogenesis, and circulating levels of β-hydroxybutyrate (βHB), acetoacetate (AcAc), and acetone. Compliance to KD can be difficult, so alternative therapies that help reduce glucose levels are needed. Exogenous ketones provide an alternative method to elevate blood ketone levels without strict dietary requirements. In this study, we tested the changes in blood glucose and ketone (βHB) levels in response to acute, sub-chronic, and chronic administration of various ketogenic compounds in either a post-exercise or rested state. WAG/Rij (WR) rats, a rodent model of human absence epilepsy, GLUT1 deficiency syndrome mice (GLUT1D), and wild type Sprague Dawley rats (SPD) were assessed. Non-pathological animals were also assessed across different age ranges. Experimental groups included KD, standard diet (SD) supplemented with water (Control, C) or with exogenous ketones: 1, 3-butanediol (BD), βHB mineral salt (KS), KS with medium chain triglyceride/MCT (KSMCT), BD acetoacetate diester (KE), KE with MCT (KEMCT), and KE with KS (KEKS). In rested WR rats, the KE, KS, KSMCT groups had lower blood glucose level after 1 h of treatment, and in KE and KSMCT groups after 24 h. After exercise, the KE, KSMCT, KEKS, and KEMCT groups had lowered glucose levels after 1 h, and in the KEKS and KEMCT groups after 7 days, compared to control. In GLUT1D mice without exercise, only KE resulted in significantly lower glucose levels at week 2 and week 6 during a 10 weeks long chronic feeding study. In 4-month and 1-year-old SPD rats in the post-exercise trials, blood glucose was significantly lower in KD and KE, and in KEMCT groups, respectively. After seven days, the KSMCT group had the most significantly reduced blood glucose levels, compared to control. These results indicate that exogenous ketones were efficacious in reducing blood glucose levels within and outside the context of exercise in various rodent models of different ages, with and without pathology.

## 1. Introduction

Glucose represents an important metabolic biomarker and is the primary fuel for most human cells. Under “normal” fed conditions with a carbohydrate-rich diet, the concentration of circulating β-hydroxybutyrate (βHB) is low, typically at <0.1 mM, and accounts for <3% of total cerebral metabolism, with minimal brain uptake [1]. However, in periods of relatively low glucose availability, such as starvation, fasting, or through the adherence of diets that reduce or restricts the ingestion of carbohydrates, such as a ketogenic diet (KD), the body shifts towards fatty acid oxidation and ketogenesis to meet metabolic demands. This fat-fueled hepatic ketogenesis elevates levels of the ketone bodies, βHB, acetoacetate (AcAc) and acetone. βHB and AcAc are converted into acetyl-CoA in the mitochondria, which enters the Krebs cycle and ensures sufficient ATP production during periods of limited glucose and glycogen availability [2,3,4]. These ketone bodies can accumulate in the blood at a combined concentration of >2 mM, and are subsequently transported across the blood brain barrier (BBB) via monocarboxylic acid transporters (MCT 1–4) to meet the brain’s metabolic demands [5].

Certain conditions, such as inflammation, oxidative stress or seizure disorders can be exacerbated by elevated blood glucose. Therefore, managing glycemia may be vital to mitigating patient risk and improving prognosis. For example, several animal studies have shown that high blood glucose levels can lead to low-grade inflammation, in addition to obesity, insulin resistance, and increased gut permeability [6]. Human studies also describe the link between high blood sugar and higher inflammatory markers. A study of 29 healthy people found that consuming only 40 g of added sugar led to an increase in inflammatory markers, while 30 min after consuming a 50 g dose of fructose, a spike in inflammatory markers, such as C-reactive protein (CRP), was described [7,8]. In another study, hyperglycemia led to an increase in the inflammatory marker Nf-κB [9].

Inflammatory responses may promote neural hyperexcitability in the brain, which leads to decreased seizure threshold in patients with seizure disorders [10]. Consequently, epileptic seizures and inflammatory mediators can form a positive feedback loop, reinforcing each other [11]. In seizure disorders, hyperglycemia is also associated with increased seizure frequency and lower seizure threshold [12,13]. Positive correlation has been described between blood sugar level and frequency and duration of seizures, while correction of hyperglycemia remains the main goal in the management of seizures [14].

Diabetes is a category of diseases resulting in glucose mismanagement and hyperglycemia [15]. Previous trials have confirmed that lowering chronic markers of glucose elevation result in improved long-term outcomes and lower incidence across common comorbidities. This effect has consistently been attributed to hyperglycemia-induced inflammation and oxidative stress, amongst others [16].

High blood glucose level can lead to further problems if it persists over a longer period of time [17,18]. In addition to inducing insulin resistance, persistent hyperglycemia impairs insulin secretion by pancreatic β-cells [19]. Chronic hyperglycemia will also cause detrimental effects on macrovascular and microvascular systems, inducing overproduction of NADH and mitochondrial reactive oxygen species (ROS) that inhibit GAPDH activity [20,21,22]. This inhibition further activates the alternative glucose metabolic pathways, which leads to increased ROS production involved in glucotoxicity that is responsible for the exacerbation of diabetes and the development of diabetic complications [22,23,24,25]. These and further studies support the concept that elevations in ROS and oxidative stress can be fomented by high blood glucose and NADH overproduction. Another recent study provides further evidence that inflammatory and oxidative stress biomarkers correlated with preclinical increases in blood glucose levels [26].

Clinically, hyperglycemia increases the risk of cerebrovascular disease, while it is also associated with increased infarct size in both myocardial infarction and stroke, increased surgical site infections, and greater severity of traumatic brain injury [27,28,29,30].

Physical exercise has also been shown to be an important mediator of glucose homeostasis [31]. Previous studies describe how physical activity influences glucose uptake, transport, and disposal [32,33,34]. It has been reported that intense exercise (VO_2max_ > 80%) leads to an eightfold increase in hepatic glucose output, while glucose utilization may increase only threefold [35,36]. In healthy individuals, insulin secretion increases during the recovery period following intense exercise to normalize plasma glucose, however this process can be impaired in diabetes, while individuals with seizure disorders are exposed to greater risk of developing a seizure in response to exercise-induced hyperglycaemia [36,37,38,39].

Improved glycemic control under baseline conditions and post-exercise can result in improved disease outcome or survival in many of the above mentioned patient populations; however, the safe reduction of blood glucose is difficult due to powerful homeostatic regulation [40]. Alternative strategies are needed to reduce blood glucose levels since medication, consistent exercise, or weight loss regimens are ineffective or difficult to maintain for many people.

While pharmacological solutions—such as metformin, insulin, SGLT2 inhibitors, and GLP inhibitors—may be used to control blood glucose levels in these populations, the issues of drug tolerance, effectiveness, compliance, and side effects can complicate the treatment in certain individuals [41,42].

A KD is a dietary strategy which promotes normoglycemia while attenuating postprandial glucose spikes. The traditional KD is composed of a 3:1 or 4:1 ratio, by weight, of fat to a combination of protein and carbohydrates that resembles some metabolic characteristics of fasting [43]. Initially, the KD was used to specifically treat epilepsy and type 1 diabetes before the development of drug therapies; however, emerging studies suggest that the KD could be a metabolic therapy for a wide range of disorders [43,44,45,46,47,48,49,50,51,52,53,54,55,56].

Despite the success of ketone-based interventions, several factors limit the efficacy and utilization of the KD as a metabolic therapy for widespread clinical use. Patient compliance to the KD can be low due to its strict requirements, individual intolerance to high-fat diets, or a general lack of knowledge and self-efficacy [57,58,59,60]. Furthermore, maintaining therapeutic ketosis can be difficult, as consumption of even a small quantity of carbohydrates or excess protein can rapidly inhibit ketogenesis [61]. Enhanced ketone body production and tissue utilization can take several weeks, and patients may experience hypoglycemic symptoms during this transitional period [62]. As such, alternative methods to rapidly establish and maintain ketosis in a patient are needed.

Previous studies have used murine models to describe changes in blood glucose and ketone levels in a rested state in response to administration of exogenous ketones [63,64,65]. However, the physiological response of glucose utilization might be different across varied physiological contexts [66,67,68,69,70,71,72,73,74,75,76,77]. Therefore, it is important to study such changes in multiple model systems typically used in metabolic studies. The effect of exogenous ketones has previously been shown on the blood glucose and ketone levels in rested non-pathological murine model, Sprague Dawley (SPD) rats, and in Wistar Albino Glaxo/Rijswijk (WAG/Rij; WR) rats. Absence epileptic activity is well-investigated in WR rats [78,79,80,81,82]. GLUT1 deficiency syndrome (GLUT1D) is a neurometabolic disorder associated with seizures, and has been studied in GLUT1 deficiency syndrome mice (GLUT 1 mice), but the effect of exogenous ketones on the blood glucose level in this animal model has not been studied yet. Patients with GLUT1D suffer from low brain glucose levels, early-onset seizures, delayed development, spasticity, ataxia, and dystonia. Therefore, it is important to find out how exogenous ketones might effect blood glucose and ketone levels in this disease [83,84,85]. In previous and the present study, the rats have been exposed to acute, sub-chronic, and chronic treatments in order to detect changes in blood glucose levels at various time points [63,86]. Several studies reported moderate or long-term effects of different composition of macronutrients in the diet, rather than the acute effects on blood kinetics [87,88,89,90]. These animal models represent an important tool for understanding the link between disease pathophysiology and glucoregulatory control.

Glucose metabolism and utilization is well known to be affected by aging [91,92,93]. Lack of adequate glucoregulatory control remains a central problem of aging and chronic disease, while numerous longevity interventions result in maintenance of glucoregulatory control [93]. To investigate the putative effect of age on exogenous ketones-induced changes in glucose levels, 4-month and 1-year-old SPD rats were studied. In this study, to further investigate the effect of KD and exogenous ketones on blood glucose and ketone (*R*-βHB) levels, we tested non-pathological (SPD rats) and pathological (WR rats and GLUT1D mice) animal models in rested and in post-exercise state acute (1 h; SPD and WR rats), sub-chronic (7 days; SPD and WR rats), and chronic treatments (10 weeks; GLUT1D mice) (Table 1).

## 2. Material and Methods

### 2.1. Animals

SPD male rats (4-months-old and 1 year old, 320–360 g and 540–660 g, respectively, Harlan Laboratories), WR male rats (6-months-old, 320–360 g, breeding colony, Eötvös Loránd University, Savaria University Centre, Szombathely, Hungary), and GLUT1D male mice (3–5-months-old, 17–27 g, breeding colony, University of South Florida (USF), Morsani College of Medicine, Tampa, FL, USA) were used. Animals were housed at either the USF College of Medicine Animal Facility, (Morsani College of Medicine, USF, Tampa, FL, USA) or the Savaria Department of Biology (Eötvös Loránd University, Savaria University Centre, Szombathely, Hungary). Animals were housed in groups of 2–4 under standard laboratory conditions (12:12 h light-dark cycle) in air-conditioned rooms at 22 ± 2 °C.

Procedures were performed in accordance with the guidelines set forth by the Institutional Animal Care and Use Committee (IACUC; Protocol #0006R) of the University of South Florida (University of South Florida, Tampa, FL, USA), the Hungarian Act of Animal Care and Experimentation (1998. XXVIII. Section 243/1998), and the regulations for animal experimentation in the European Communities Council Directive of 24 November 1986 (86/609/EEC). All experiments were approved, and all efforts were made to reduce the number of animals used.

The experimental design was approved by the Animal Care and Experimentation Committee of the Eötvös Loránd University (Savaria University Centre) and National Scientific Ethical Committee on Animal Experimentation (Hungary) under license number VA/ÉBNTF02/85-8/2016.

### 2.2. Diets and Ketogenic Compounds

Animals were allowed ad libitum access to water and standard rodent chow (SD, 2018 Teklad Global 18% Protein Rodent Diet; #2018, Harlan), ketogenic rodent food (KD, Table 2), or SD mixed with ketone supplementation.

The ketone ester (KE) *R*, *S* 1, 3-butanediol-acetoacetate diester was synthesized as previously described by D’Agostino et al. [94]. The ketone salt Na^+^/K^+^– *R*, *S* βHB mineral salt (KS) is a novel agent that was mixed into a 50% solution, supplying approximately 375 mg/g of pure *R*-βHB and 125 mg/g of Na^+^/K^+^ in a 1:1 ratio. Both KE and KS were developed and synthesized in collaboration with Savind Inc. Human food-grade medium chain triglyceride (MCT) oil (~60% caprylic triglyceride/40% capric triglyceride) was purchased from Now Foods (Bloomingdale, IL, USA). KS or KE were mixed with MCT in a 1:1 ratio, generating the KSMCT and KEMCT combinations. KE was mixed with KS in a 1:1 ratio to create KEKS. R, S-1, 3-butanediol (BD) was purchased from Sigma (Milwaukee, WI, USA).

### 2.3. Treatment Groups

**Figure 1:**
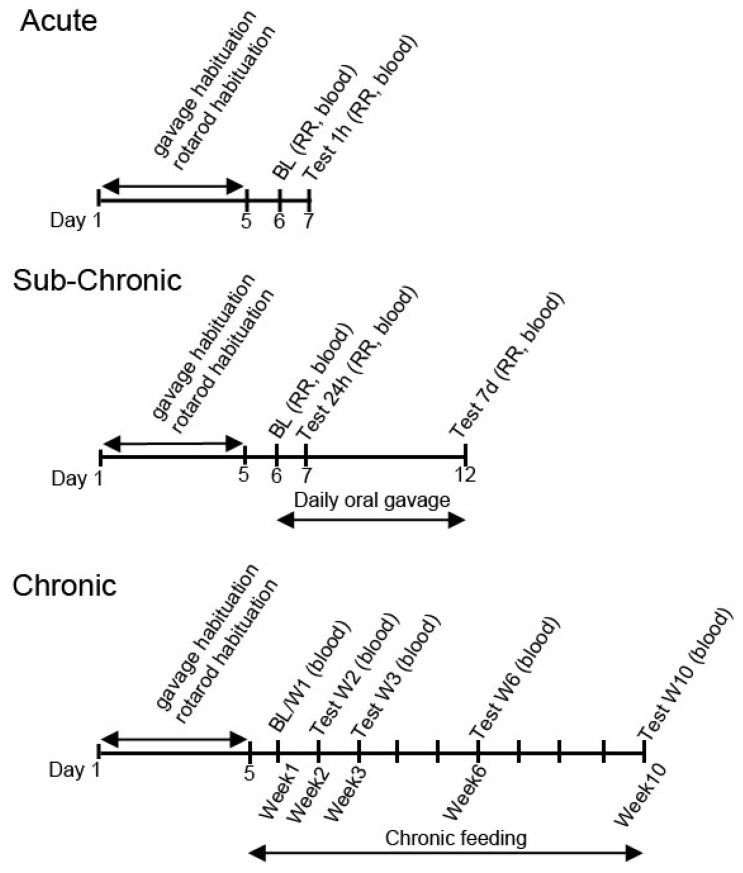
Schematic drawing of the experimental design. Abbreviations: BL: Baseline measurement; RR: rotarod.

To habituate the rodents to intragastric delivery, animals were orally gavaged with water for five days prior to treatment (Figure 1). After habituation and baseline measurements (on the 5th day of habituation), the rodents were orally gavaged either once with exogenous ketones (acute treatment; 5 g/kg for SPD rats and 2.5 g/kg/day for WR rats) and the effect was measured after 1 h, or they were gavaged once daily for 7 days (sub-chronic treatment; 5 g/kg/day for SPD rats and 2.5 g/kg/day for WR rats) and the effect on blood glucose and ketones was recorded after 24 h and after 7 days (Figure 1).

For the acute treatment on 1-year-old SPD rat experiment with exercise, the treatment groups included water (control, *n* = 10), BD (*n* = 8), KE (*n* = 12), KSMCT (*n* = 8), KEKS (*n* = 12), and KEMCT (*n* = 8). For the sub-chronic treatment on 4-month-old SPD rats experiment with exercise, the treatment groups included control (*n* = 11), KD (*n* = 10), KE (*n* = 9), KS (*n* = 9), and KSMCT (*n* = 10) while on standard diet (SD). For acute and sub-chronic experiments on rested 6-month-old WR rats, the rodents were orally gavaged with either water (SD: control, *n* = 9), KE (*n* = 9), KS (*n* = 9), or KSMCT (*n* = 9) while on SD. For the exercised WR experiments, the rodents were fed either a SD (*n* = 9) or a diet supplemented with either KE (*n* = 9), KS (*n* = 9), KSMCT (*n* = 9), KEKS (*n* = 9), or KEMCT (*n* = 9). The GLUT1D mice were fed for 10 weeks (chronic treatment) on either a ketogenic diet (KD, *n* = 12), SD (*n* = 12), or the SD supplemented with 20% KS (*n* = 12) or 10% KE (*n* = 12).

### 2.4. Exercise with Accelerated Rotarod

For all trials involving exercise (SPD or WR rats), the rodents were exercised on a rotarod Rotamex 5 (Columbus Instruments, Columbus, OH, USA). The animals were trained on the rotarod for five consecutive days before treatment began to acclimate them to the equipment and the task (habituation to rotarod test was parallel with habituation to oral gavage; Figure 1). To evaluate exercise-induced fatigue, the rotarod was set to accelerate from zero to 40 rpm over a protracted period of 180 s for all training periods and trials, across all experiments. Each session of training and testing consisted of three trials, with a two-minute rest period between each trial. Blood measurements were collected within 10 min after last trial.

### 2.5. Measurement of Blood R-βHB and Glucose

Whole blood samples (~10 μL) were taken from the saphenous vein of rats and from the tail vein of mice for analysis of blood glucose (mg/dL) and *R*-βHB (mmol/L) levels using a commercially available glucose and ketone monitoring system, Precision Xtra^TM^ (Abbott Laboratories, Abbott Park, IL, USA). Note that the Precision Xtra^TM^ only measures *R*-βHB levels—not *S*-βHB, AcAc or Acetone—therefore, total blood ketone levels may be higher than measured. For all experiments, blood was initially drawn prior to the beginning of the intervention (on the 5th day of habituation), with this value used as the established baseline.

Blood was drawn after treatment was started either 1 h, 24 h, or after 7 days (Figure 1). In exercised trials, blood was drawn within 10 min after last trial was completed. During chronic treatment, blood was drawn at week 1 before treatment started (baseline), and at week 2, week 3, week 6, and at week 10 after the beginning of the intervention.

### 2.6. Statistics

All data is presented as the mean ± standard error of the mean (SEM). The effects of ketogenic compounds on blood *R*-βHB and glucose levels were compared to experimental controls and respective baseline levels. Data analysis was performed using GraphPad Prism version 6.0a. Blood ketone and glucose levels were compared using a one or two-way ANOVA with Tukey’s multiple comparisons test. Results were considered significant when p values were less than 0.05. Results are indicated on figures using the following notations: *-*p* < 0.05, **-*p* < 0.005, ***-*p* < 0.0005, or ****-*p* < 0.0001.

## 3. Results

### 3.1. Acute Effect of Ketone Supplements on Blood Glucose and R-βHB Levels in Exercised Sprague-Dawley Rats

**Figure 2:**
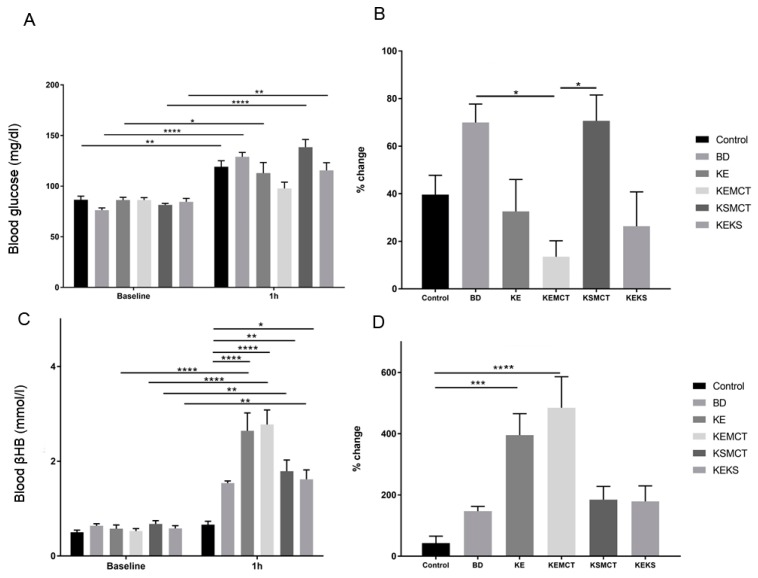
Changes in blood glucose and *R*-βHB levels of 1-year-old SPD rats after 1 h of treatment, in post-exercise state. (**A**) In KEMCT group the blood glucose level was not significantly elevated, compared to baseline. (**B**) The corresponding percent change in glucose levels. (**C**) The resulting blood *R*-βHB levels. (**D**) The percent change in the blood *R*-βHB levels. Abbreviations: SPD: Sprague-Dawley rat; BD: 1, 3-butanediol; KE: ketone ester; KEMCT: ketone ester and medium chain triglyceride, 1:1 ratio; KSMCT: ketone salt and medium chain triglyceride, 1:1 ratio; KEKS: ketone ester and ketone salt, 1:1 ratio. *: *p* < 0.05, **: *p* < 0.01, ***: *p* < 0.001 and ****: *p* < 0.0001 level of significance.

The effect of different combinations of exogenous ketone supplements on blood glucose levels following a single gavage administration (acute treatment) in an exercised state was first tested in one-year-old SPD rats (Figure 2). After 1 h, in all treatment groups (Control: *p* = 0.0064; BD: <0.0001; KE: *p* = 0.025; KSMCT: *p* < 0.0001; KEKS: *p* = 0.0048), except in KEMCT, the blood glucose levels were significantly elevated, compared to their baseline level (Figure 2A). Depending on the group, the blood drawn from the rats 1 h after administration of supplements, although insignificant, showed either a trend of decreased (KE, KEMCT, and KEKS groups) and increased (BD and KSMCT groups) percent change in blood glucose levels when compared to the control group (Figure 2B). However, the percent change in blood glucose levels in KEMCT group was significantly lower than in BD (*p* = 0.0364) and in KSMCT (*p* = 0.0328), respectively. The rats also showed significantly higher levels of blood *R-*βHB levels after exercise compared to baseline in all treatment groups, except in BD, compared to the control group (KE: *p* < 0.0001; KEMCT: *p* < 0.0001; KSMCT: *p* = 0.004; KEKS: *p* = 0.0119) and their respective baseline (KE: *p* < 0.0001; KEMCT: *p* < 0.0001; KSMCT: *p* = 0.0099; KEKS: *p* = 0.0021) with the KE and KEMCT groups resulting in the highest increase in blood ketone levels (Figure 2C). The percent change in blood R-βHB levels was significantly higher in KE (*p* = 0.0005) and KEMCT (*p* < 0.0001) groups, compared to control (Figure 2D).

### 3.2. Sub-Chronic Effect of Ketone Supplements on Blood Glucose and R-βHB Levels in Exercised Sprague-Dawley Rats

**Figure 3:**
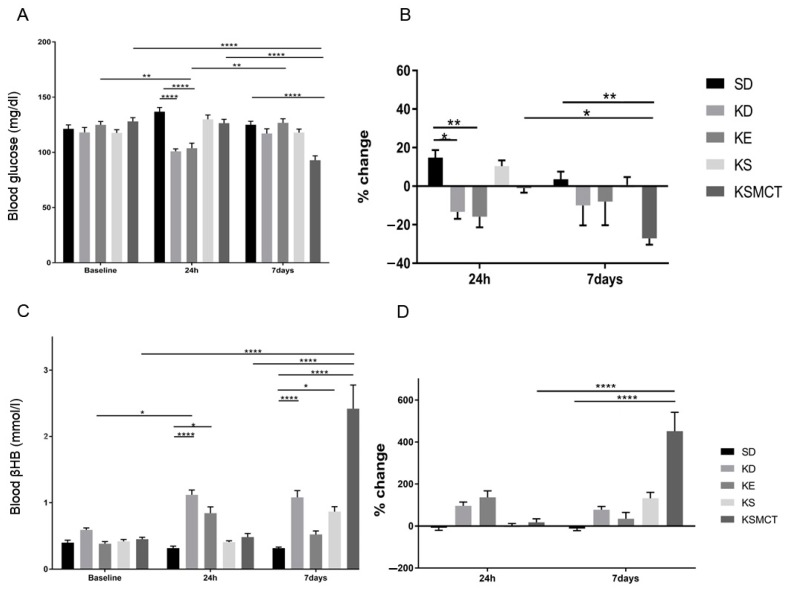
Changes in blood glucose and *R*-βHB levels of 4-months-old SPD rats after 24 h and seven days of treatment, in post-exercise state. (**A**) The change in glucose levels in SPD rats, with exercise, for the baseline, 24 h, and seven days post-intervention. (**B**) The corresponding percent change in glucose levels. (**C**) The resulting blood *R*-βHB levels. (**D**) The percent change in the blood *R*-βHB levels. Abbreviations: SPD: Sprague-Dawley rat; SD: standard diet (control); KD: ketogenic diet; KE: ketone ester; KS: ketone salt; KSMCT: ketone salt and medium chain triglyceride, 1:1 ratio. *: *p* < 0.05, **: *p* < 0.01, and ****: *p* < 0.0001 level of significance.

The ability of a KD or exogenous ketones to reduce blood glucose levels following sub-chronic gavage administration and rotarod training was tested in 4-month-old SPD rats. The animals were fed either with SD or KD diet, or SD combined with daily gavage administration of ketone supplements for seven days, while blood levels were measured after 24 h and after seven days of treatment (Figure 3). Rats fed a KD (*p* < 0.0001) or SD with KE supplement (*p* < 0.0001) had significantly decreased levels of blood glucose after 24 h, compared to control (Figure 3A). At the end of the seven-day treatment, only the KSMCT group had a significantly lower level of blood glucose, compared to control group (*p* < 0.0001). KE group had lower blood glucose levels at 24 h (*p* = 0.0047), compared to its baseline. However, after seven days the level increased in this group, compared to the 24 h level (*p* = 0.0049). KSMCT was significantly lower after seven days, compared to its baseline (*p* < 0.0001), compared to the 24 h level (*p* < 0.0001), and compared to the control group (*p* < 0.0001; Figure 3A). The percent change of blood glucose level was significantly decreased in KD (*p* = 0.02) and KE (*p* = 0.007) groups after 24 h, compared to control (Figure 3B). The percent change of blood glucose level in KSMCT was significantly lower at day 7 than at 24 h (*p* = 0.041) and the control group at day 7 (*p* = 0.007) (Figure 3B).

Rats fed a KD exhibited an increase in *R*-βHB after 24 h (*p* = 0.038), compared to baseline, and compared to control at 24 h (*p* < 0.0001), while rats given SD supplemented with KE showed elevated levels at 24 h (*p* = 0.0325), compared to control (Figure 3C). Rats fed a KD (*p* < 0.0001), SD supplemented with KS (*p* = 0.0194), and KSMCT (*p* < 0.0001) showed a significant increase in *R*-βHB levels after seven days, compared to control. KSMCT also showed a significant increase, compared to its baseline (*p* < 0.0001) and the 24 h level (*p* < 0.0001). The percent change in *R*-βHB levels was significantly higher only in KSMCT at day 7, compared to 24 h (*p* < 0.0001), and compared to control group (*p* < 0.0001) at seven days (Figure 3D).

### 3.3. Acute Effect of Ketone Supplements on Blood Glucose and R-βHB Levels in Rested WAG/Rij Rats

**Figure 4:**
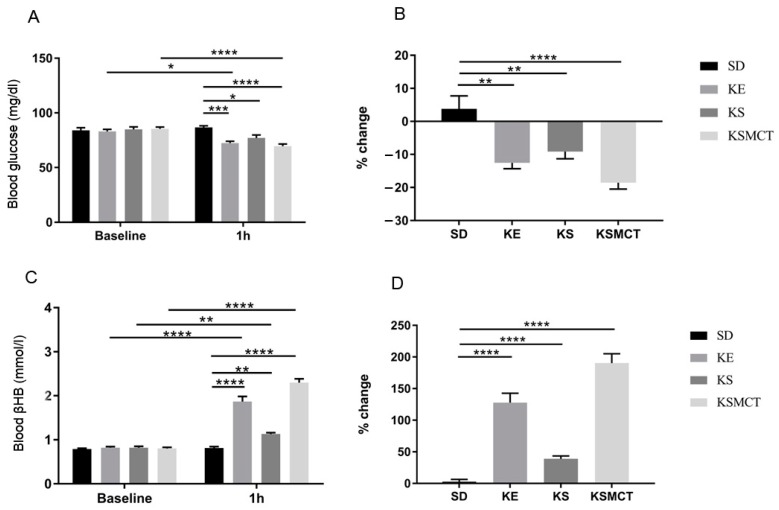
Changes in blood glucose and *R*-βHB levels of 6-months-old WR rats after 1 h of treatment, in rested state. (**A**) The change in glucose levels in WR rats, with no exercise, for the baseline and 1 h post-treatment. (**B**) The corresponding percent change in glucose levels. (**C**) The resulting blood *R*-βHB levels. (**D**) The percent change in the blood *R*-βHB levels. Abbreviations: WR: WAG/Rij rat; SD: standard diet (Control); KE: ketone ester; KS: ketone salt; KSMCT: ketone salt and medium chain triglyceride, 1:1 ratio. *: p<0.05, **: *p* < 0.01, ***: *p* < 0.001 and ****: *p* < 0.0001 level of significance.

WR rats were tested in acute and sub-chronic conditions, and in both a post-exercise and rested states with exogenous ketone supplements. In trials without exercise, following acute exposure (1 h after oral gavage), all ketone supplemented treatment groups had significant reductions in blood glucose levels and significant increases in blood ketone levels, when compared to the control (Figure 4). The most significant reduction in blood glucose was in KSMCT group, compared to control (*p* < 0.0001), and compared to baseline (*p* < 0.0001; Figure 4A). The elevation in blood βHB was most significant in KE and KSMCT treatment groups, compared to control (*p* < 0.0001) and to baseline (*p* < 0.0001; Figure 4C). Reduction in blood glucose levels could be observed in KE group, compared to its baseline (*p* = 0.0119), and compared to control (*p* = 0.0002) at 1 h (Figure 4A). KS and KSMCT caused lowered blood glucose at 1 h, compared to control (*p* = 0.0322 and *p* < 0.0001, respectively). The percent change in blood glucose levels showed reduction in KE (*p* = 0.0005), KS (*p* = 0.0068), and KSMCT (*p* < 0.0001) groups as well (Figure 4B). All treatment groups had significantly elevated *R*-βHB levels after 1 h, compared to their baseline (KE: *pp* < 0.0001; KS: *p* = 0.0053; KSMCT: *p* < 0.0001) and to control (KE: *p* < 0.0001; KS: 0.0034; KSMCT: *p* < 0.0001; Figure 4C). The percent change in blood *R*-βHB levels showed increase in all treatment groups (*p* < 0.0001; Figure 4D).

### 3.4. Sub-Chronic Effects of Ketone Supplements on Blood Glucose and R-βHB Levels in Rested WAG/Rij Rats

**Figure 5:**
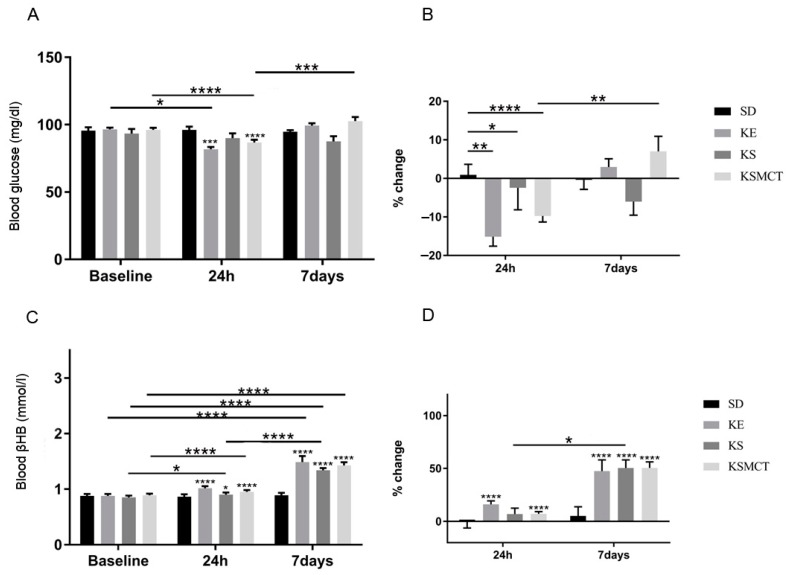
Changes in blood glucose and *R*-βHB levels of 6-month-old WR rats after 24 h and seven days of treatment, in rested state. (**A**) The change in glucose levels in WR rats, with no exercise, for the baseline, after 24 h and after seven days of daily treatment. (**B**) The corresponding percent change in glucose levels. (**C**) The resulting blood *R*-βHB levels. (**D**) The percent change in the blood *R*-βHB levels. Abbreviations: WR: WAG/Rij rat; SD: standard diet (Control); KE: ketone ester; KS: ketone salt; KSMCT: ketone salt and medium chain triglyceride, 1:1 ratio. *: *p* < 0.05, **: *p* < 0.01, ***: *p* < 0.001 and ****: *p* < 0.0001 level of significance.

The effect of sub-chronic exposure of ketone supplementation by oral gavage in WR rats on blood glucose and *R*-βHB levels were documented after 24 h and seven days, without exercise (Figure 5). Blood glucose levels decreased in KE group compared to its baseline (*p* = 0.029) and to control (*p* = 0.0004) after 24 h (Figure 5A). At 24 h, KSMCT as well had lower blood glucose levels, compared to control (*p* < 0.0001). The blood glucose level in KSMCT was lower at 24 h, compared to its baseline (*p* < 0.0001), but higher at seven days, compared to the level at 24 h (*p* = 0.001). The percent change of blood glucose level showed a significant decrease in all treatment groups at 24 h (KE: *p* = 0.0026; KS: *p* = 0.0353; KSMCT: *p* < 0.0001; Figure 5B). Blood *R*-βHB levels were elevated at 24 h in all treatment groups, (KE: *p* < 0.0001; KS: *p* = 0.02; KSMCT: *p* < 0.0001) and after seven days (*p* < 0.0001), compared to control (Figure 5C). All treatment groups had increased *R*-βHB levels at seven days, compared to their baseline (*p* < 0.0001). KS had elevated *R*-βHB level at 24 h, compared to its baseline level (*p* = 0.0294) and at seven days, compared to the level at 24 h (*p* < 0.0001). The percent change of *R*-βHB level was higher than control in KE and KSMCT at 24 h (*p* < 0.0001), while it was higher than control in all treatment groups at seven days (*p* < 0.0001; Figure 5D). The percent change of *R*-βHB level was higher in KS group at seven days than at 24 h (*p* = 0.0122).

### 3.5. Acute and Sub-Chronic Effects of Ketone Supplements on Blood Glucose and R-βHB Levels in Exercised WAG/Rij Rats

**Figure 6:**
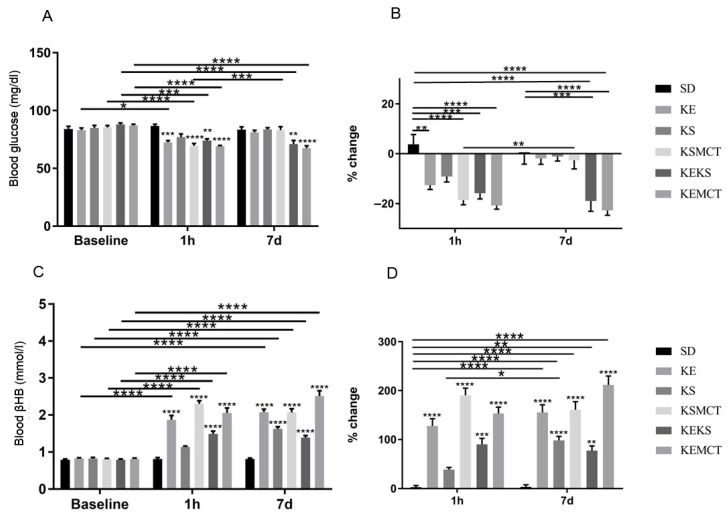
Changes in blood glucose and *R*-βHB levels of 6-month-old WR rats after 1 h and seven days of treatment, in post-exercise state. (**A**) The change in glucose levels in WR rats, with exercise, for the baseline, 1 h and seven days of the treatment. (**B**) The corresponding percent change in glucose levels. (**C**) The resulting blood *R*-βHB levels. (**D**) The percent change in the blood *R*-βHB levels. Abbreviations: WR: WAG/Rij rat; SD: standard diet (Control); KE: ketone ester; KS: ketone salt; KSMCT: ketone salt and medium chain triglyceride, 1:1 ratio; KEKS: ketone ester and ketone salt, 1:1 ratio; KEMCT: ketone ester and medium chain triglyceride, 1:1 ratio. *: *p* <0.05, **: *p* <0.01, ***: *p* <0.001 and ****: *p* < 0.0001 level of significance.

Exercised WR rats were examined at 1 h and seven days after exogenous ketone treatment (Figure 6). All treatment groups had lower blood glucose levels at the 1 h mark (KE: *p* = 0.0002; KSMCT: *p* < 0.0001; KEKS: *p* = 0.022; KEMCT: *p* < 0.0001), except KS, compared to control, and compared to their baseline (KE: *p* = 0.03; KSMCT: *p* < 0.0001; KEKS: *p* = 0.0003; KEMCT: *p* < 0.0001; Figure 6A). Blood glucose was lower only in KEKS (*p* = 0.0034) and KEMCT (*p* < 0.0001) groups at seven days, compared to control and compared to their baseline (*p* < 0.0001). KSMCT had increased glucose level at seven days compared to the level at 24 h (*p* = 0.0006). The percent change of blood glucose levels decreased at 1 h in all treatment groups (KE: *p* = 0.0033; KSMCT: *p* < 0.0001; KEKS: *p* = 0.0001; KEMCT: *p* < 0.0001), except in KS, compared to control, and decreased only in KEKS (*p* = 0.0004) and KEMCT (*p* < 0.0001) at seven days, compared to control (Figure 6B). The percentage change of blood glucose was significantly reduced in KEKS (*p* < 0.0001) and KEMCT (*p* < 0.0001) groups compared to their baseline as well, while KSMCT was lower at seven days than at 1 h (*p* = 0.0048).

All treatment groups had significantly elevated blood *R*-βHB levels at 1 h (*p* < 0.0001), except KS, and after 7 days (*p* < 0.0001), compared to control (Figure 6C). Also, all treatment groups had elevated *R*-βHB levels at 1 h (*p* < 0.0001), except KS, and at seven days (*p* < 0.0001), compared to their baseline. The percent change of blood *R*-βHB was elevated in all, except in KS at 1 h (*p* < 0.0001; KEKS: *p* = 0.0002) and was elevated in all treatment groups at seven days (*p* < 0.0001, KEKS: *p* = 0.0029), compared to control (Figure 6D). Also, all treatment groups were elevated at seven days (*p* < 0.0001, KEKS: *p* = 0.0026), compared to the control at 1 h (*p* < 0.0001). The *R*-βHB level in KS was higher at seven days than at 1 h (*p* = 0.0421).

### 3.6. Chronic Effects of Ketone Supplements on Blood Glucose and R-βHB Levels in G1D Syndrome Mice

**Figure 7:**
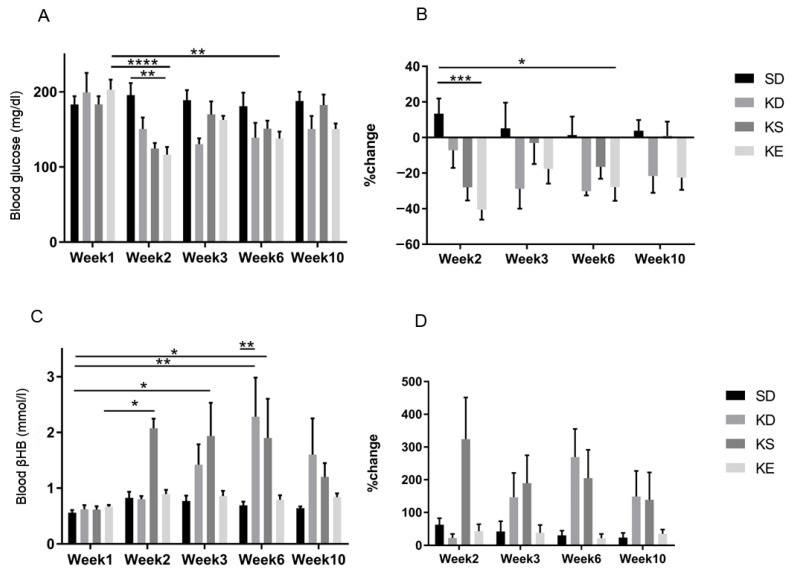
The effect of chronic feeding of ketogenic compounds on blood glucose and *R*-βHB level was assessed in glucose transporter type 1 (G1D)-deficiency syndrome mice during 10 weeks long experiment, in rested state. (**A**) The change in blood glucose levels in GLUT1D mice, without exercise, chronically exposed to various ketone supplements. (**B**) The corresponding percent change in blood glucose levels. (**C**) The resulting blood *R*-βHB levels. (**D**) The percent change in blood *R*-βHB levels. Abbreviations: SD: standard diet (Control); KD: ketogenic diet; KS: ketone salt; KE: ketone ester. *: *p* < 0.05, **: *p* < 0.01, ***: *p* < 0.001 and ****: *p* < 0.0001 level of significance.

The effect of chronic feeding of KD or exogenous ketone supplements on blood glucose levels was assessed in GLUT1D mice for 10 weeks (Figure 7). There was a trend of lower blood glucose levels in every treatment group at every week after treatment started, however, the differences reached significance only at a few time points (Figure 7A). The KE group had the largest reduction in blood glucose at week 2 (*p* = 0.0017), compared to control, and to baseline, week 1 (*p* < 0.0001), while it was significantly lower at week 6 (*p* = 0.0098), compared to its baseline (Figure 7A). The percent change in blood glucose levels was significantly decreased at week 2 in KE (*p* = 0.0004) and at week 6 (*p* = 0.024), compared to the baseline control level (Figure 7B).

The KS treatment caused a significant and rapid elevation of blood ketone levels at week 2 (*p* = 0.0342), week 3 (*p* = 0.0215), and at week 6 (*p* = 0.0161), compared to control group at week 1. The KD group had a slow, but constant increase in blood *R*-βHB levels, reaching significance at week 6 (*p* = 0.0017), compared to control group at week 1. KE caused consistent, but slight elevation in blood *R*-βHB levels, compared to its baseline, however it did not reach significance (Figure 7C,D).

## 4. Discussion

These results demonstrate the blood glucose lowering effect of the ketogenic diet and ketone supplements in SPD and WR rats, as well as in GLUT1D mice, after acute, sub-chronic, or chronic administrations. These murine model systems are frequently used in studies where therapeutic ketosis and glucoregulatory control are important influencers of disease management or prevention of symptoms. The glucoregulatory effects of ketone supplementation was variable between treatment groups (rested and post-exercise state), suggesting that the different physiological states influence ketone-induced alterations in blood glucose levels. The results confirm and extend our previously reported results of decreased blood glucose in SPD and WR rats receiving ketone supplements, and were also extended to GLUT1D mice [63]. The exogenous ketone-induced blood glucose lowering effects in rats varied depending on the strain, administration, the type of supplement, age, and exercise state.

In a previous study in juvenile SPD rats, we found no significant change in the baseline blood glucose or ketone levels after 4-week gavage [63]. However, blood glucose levels were reduced after acute gavage administration with KSMCT and MCT groups. KS significantly lowered blood glucose only at 8 h/week 1 and 12 h/week 3. Significantly reduced blood glucose levels were observed in KE group, compared to controls between weeks 1–4. BD did not have a significant effect on blood glucose levels at any time point during the 4-week study.

Glucose production and utilization can change with age, therefore we tested different age groups of rodents [93]. During exercise, the control of glucose homeostasis is dictated by a complex interaction between multiple hormonal regulators (e.g., insulin, glucagon, catecholamines, and glucocorticoids), the nervous system, and various molecular regulators within skeletal muscle and liver, that maintain precise control of glucose concentration during most activities. In order to better understand the glucose homeostasis during exercise, we used the rotarod exercise to simulate post-exercise state in murine models. During the present study, in exercised 1-year-old SPD rodents, after acute treatment, all treatment groups had increased blood glucose levels, except in the KEMCT group. In exercised 4-months-old SPD rats with sub-chronic exposure, at 24 h of intervention, the KD and KE groups had significantly lower glucose levels, while these same groups had significantly higher *R*-βHB levels. It is conceivable that higher doses would have decreased blood glucose and increased *R*-βHB levels in the remaining groups, but this needs further validation. However, after seven days of treatment, only KSMCT had a significant reduction in blood glucose and significant increase in βHB, implying that short-term and long-term use of various ketone supplements may have different effects on blood glucose. In acutely exposed WR rats, without exercise, all groups had significantly reduced glucose levels compared to the control, while all treatments increased *R*-βHB levels. In the sub-chronically treated WR rats without exercise, after 24 h, only KE and KSMCT lowered blood glucose significantly, while KE, KS, and KSMCT increased *R*-βHB significantly. After seven days of treatment, none of the treatment groups had significantly lower glucose levels, while all treatments caused a significant increase in *R*-βHB levels. It is also possible that a higher dose would be more effective to achieve the blood glucose lowering effect, but this would be approaching the maximum tolerable levels. In WR rats, with exercise, all groups had a significant reduction in blood glucose levels and significant increases in *R*-βHB after one hour, except KS. However, after sub-chronic (seven days) exposure, only the KEKS and the KEMCT treatments reduced blood glucose significantly, while all treatments significantly elevated βHB. For the GLUT1D mice with a chronic 10-week exposure schedule, KE significantly reduced blood glucose at two weeks and six weeks. The blood ketone levels were not elevated significantly in most cases (suggesting greater ketone utilization), therefore higher doses might be used in the future to more effectively lower blood glucose levels and elevate blood ketone levels in this animal model.

Regarding age, while KD, KE, and KSMCT decreased glucose in exercised young adult SPD rats after 1 h, it was ineffective in the older (1-year-old) SPD rat cohort after 24 h; KEMCT was the only supplement that didn‘t cause elevated blood glucose in the older animals. Interestingly, in rats with pathology (WR) after acute treatment and exercise, the blood glucose level was lower in KE, KSMCT, KEKS, and KEMCT treatment groups compared to control, further supporting the hypothesis that age and pathological state might influence the bodies’ response to nutritional supplements. Rested GLUT1D mice, which is a model of human GLUT1D, exhibited a sustained, although not significant, decrease in blood glucose levels over several weeks when consistently given ketone supplements [95]. Based on these results, we can speculate that there are differences in ketone-induced lowering of blood glucose between the various age groups and pathologies. The mechanisms of action may change as the organism ages. However more mechanistic studies are needed that focus specifically on the effect of aging on glucose disposal and hepatic gluconeogenesis.

A KD replicates some aspects induced by fasting, including a reduction in glucose fluctuations, and is frequently used to treat drug-resistant seizures [48]. Efficacy of KD has been positively correlated to the levels of circulating ketone bodies [96], however, using this dietary therapy can still be problematic for many patients.

Recent studies using ketone esters of βHB or AcAc have shown they are effective in inducing rapid and sustained ketosis, and that they are safe and well-tolerated in rats and humans [94,97,98]. Previously, we have reported successful use of KE in studies on tumor proliferation, central nervous system oxygen toxicity, and absence epileptic activity [53,80,94]. In this report, we present data showing that ketone supplementation may represent an alternative strategy to control blood glucose levels.

By far the most prevalent disorder of hyperglycemia is diabetes mellitus (DM), comprised of both insulin-dependent (type 1 or IDDM) and non-insulin-dependent (type 2 or NIDDM), with type 2 diabetes making up the majority of cases, especially in the western world. Glucose toxicity is the primary cause of most diabetic vascular complications, and strong glycemic control can significantly improve patient outcomes [99]. Interestingly, in addition to treating epilepsy, KD was also the standard treatment for DM until the advent of insulin treatment [44]. Recently it has been reported that in Type-1 and Type-2 diabetic patients, a low-carbohydrate, KD results in improved glycemic control [56,100,101].

Other clinically relevant states in which glycemic control is compromised include traumatic injury and post-surgical recovery, in which elevated blood glucose levels associated with poorer outcomes in each [29,30]. Currently the mechanism of glucose toxicity is unclear, but strict glycemic control is associated with improved outcomes in critically ill patients [40]. The ability to improve glycemic control in patients via a dietary supplement, such as exogenous ketone supplementation, could be advantageous, since it may help to reduce over-dependence on aggressive insulin therapy [102]. Further studies are needed in order to determine whether exogenous ketone supplements could improve glycemic control and provide a beneficial adjunct to these patients.

These results, taken together, indicate that the ketone-induced ability to acutely lower blood glucose is likely present, even in post-exercise state, and likely has different mechanisms based on the type of ketogenic formulation and disease pathology. The observation that KE reduced glucose levels in exercised SPD rats in this study is consistent with previously reported results in non-exercised SPD rats [86].

Some have suspected that a ketone induced elevation of insulin may be mediating the glucose-lowering effect of exogenous ketones, especially if given acutely as a large dose [103], although an increase in insulin sensitivity could also be a factor [104]. However, it has also been demonstrated that βHB-infusion in Type-1 Diabetic children resulted in significant reductions in blood glucose, suggesting exogenous βHB may lower BG independent of endogenous insulin secretion [105].

The availability of ketone bodies as alternative fuels for neuronal metabolism is postulated to be the mechanism of the therapeutic effect of KD on GLUT1DS [106]. It is reasonable to predict that ketone supplements would provide a similar effect on this neurometabolic disorder by elevating blood ketones. In addition to future functional and behavioral tests in GLUT1D mice, it will be important to determine if there are ketone-induced changes in GLUT1D cerebrospinal fluid glucose levels.

Overall, these results confirm the previous observation of ketone supplements reducing blood glucose levels [63,86]. We report that exogenous ketones can be used to reduce blood glucose and elevate blood ketone levels effectively to a variable degree, in a variety of pathological and non-pathological rodent models, in both rested and post-exercise states, across age groups. These results support the conclusion that exogenous ketone supplements have potential value in inducing therapeutic ketosis and reducing blood glucose levels. Further studies are needed to elucidate the ketone-induced glucoregulatory mechanism these compounds have, and if the benefits can be extended to humans.

## Figures and Tables

**Table 1:** Treatment groups in the different rodent models.

	Acute	Sub-Chronic	Chronic
**SPD**	(R)Ex	(R)Ex	(R)
**WR**	R, Ex	R, Ex	
**GLUT1D**			R

Ex: exercised, R: Rested, SPD: Sprague-Dawley rats, WR: WAG/Rij rats, GLUT1D: GLUT1D deficiency syndrome mice, (R): Rested state in parenthesis indicates that data is already described in earlier literature.

**Table 2:** Macronutrient ratios of rodent standard diet and ketogenic diet used.

Macronutrient Information	Standard Diet (SD)	Ketogenic Diet (KD)
% Cal from Fat	18.0	77.1
% Cal from Protein	24.0	22.4
% Cal from Carbohydrate	58.0	0.5
Caloric Density (Kcal/g)	3.1	4.7

## Abbreviations

βHB	beta-hydroxybutyrate
GLUT1D	GLUT1 deficiency syndrome
KD	ketogenic diet
KE	1, 3 butanediol-acetoacetate diester
KS	ketone salt
SD	standard diet
SPD rats	Sprague-Dawley rats
WAG/Rij	Wistar Albino Glaxo/Rijswijk rats
	
